# COVID-19 vaccine antibody responses in community-dwelling adults to 48 weeks post primary vaccine series

**DOI:** 10.1016/j.isci.2023.106506

**Published:** 2023-03-28

**Authors:** Sharon L. Walmsley, Leah Szadkowski, Bradly Wouters, Rosemarie Clarke, Karen Colwill, Paula Rochon, Michael Brudno, Rizanni Ravindran, Janet Raboud, Allison McGeer, Amit Oza, Christopher Graham, Amanda Silva, Dorin Manase, Peter Maksymowsky, Laura Parente, Roaya Monica Dayam, Jacqueline Simpson, Adrian Pasculescu, Anne-Claude Gingras

**Affiliations:** 1 Department of Medicine, University Health Network, University of Toronto, Toronto, ON, Canada; 2 Biostatistics Research Unit, University Health Network, University of Toronto, Toronto, ON, Canada; 3 Toronto General Hospital Research Institute, University of Toronto, Toronto, ON, Canada; 4 Princess Margaret Cancer Center, University of Toronto, Toronto, ON, Canada; 5 Lunenfeld-Tanenbaum Research Institute, Mount Sinai Hospital, University of Toronto, Toronto, ON, Canada; 6 Women’s College Hospital Research Institute, University of Toronto, Toronto, ON, Canada; 7 Department of Computer Science, University Health Network, University of Toronto, Toronto, ON, Canada; 8 Dalla Lana School of Public Health, University of Toronto, Toronto, ON, Canada; 9 Department of Medicine, Mount Sinai Hospital, University of Toronto, Toronto, ON, Canada; 10 Trillium Health Partners, Department of Medicine, University of Toronto, Toronto, ON, Canada; 11 Department of BioInformatics, University Health Network, University of Toronto, Toronto, ON, Canada; 12 Health Care Human Factors, University Health Network, University of Toronto, Toronto, ON, Canada; 13 University of Toronto, Toronto, ON, Canada

**Keywords:** Health sciences, Public health, Immunology, Immune response, Microbiology

## Abstract

We report a decentralized prospective cohort study of self-reported adverse events and antibody responses to COVID vaccines derived from dried blood spots. Data are presented for 911 older (aged >70 years) and 375 younger (30–50 years) recruits to 48 weeks after the primary vaccine series. After a single vaccine, 83% younger and 45% older participants had overall seropositivity (p < 0.0001) increasing to 100/98% with the second dose, respectively (p = 0.084). A cancer diagnosis (p = 0.009), no mRNA-1273 vaccine doses (p <0 .0001), and older age (p <0 .0001) predicted lower responses. Antibody levels declined in both cohorts at 12 and 24 weeks increasing with booster doses. At 48 weeks, for participants with 3 vaccine doses, the median antibody levels were higher in the older cohort (p = 0.04) with any dose of mRNA-1273 (p <0 .0001) and with COVID infection (p <0 .001). The vaccines were well tolerated. Breakthrough COVID infections were uncommon (16% older cohort, 29% younger cohort; p < 0.0001) and mild**.**

## Highlights

• Seroconversion rates were low in the older participants after one COVID vaccine
• Most participants seroconverted after the two doses of vaccine
• 48 weeks after two vaccines, median antibody levels were higher in the older cohort
• Higher antibody levels were also seen with COVID infection and mRNA-1273 vaccine

## Graphical abstract

**Figure:**
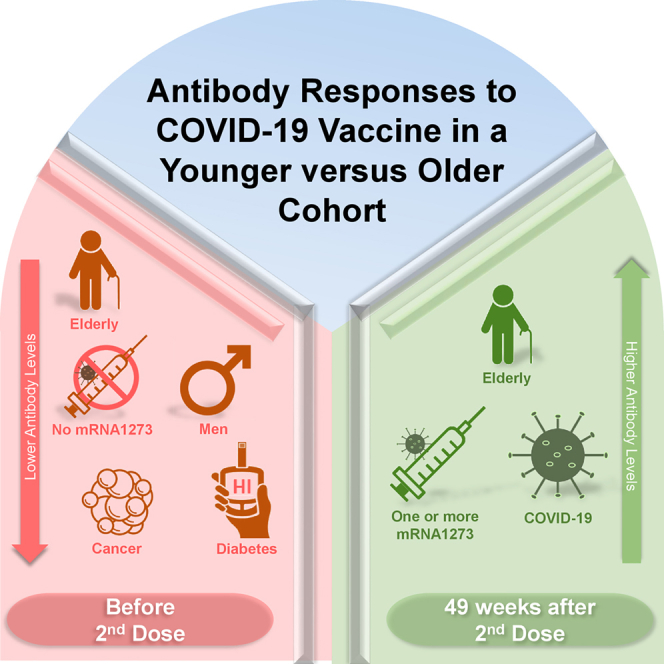

## Introduction

Clinical trials and population-based studies demonstrate good efficacy and safety profiles for COVID-19 mRNA vaccines.^1^^,^^2^^,^^3^^,^^4^^,^^5^^,^^6^^,^^7^^,^^8^ Older persons, especially those with comorbidity, have higher mortality from COVID-19 infection and were prioritized for vaccination in most countries. Antibodies resulting from natural infection appear partially protective against re-infection^9^^,^^10^ and disease severity.^11^ We remain unable to define an immunity threshold for COVID-19 vaccines that confers protection against infection,^12^^,^^13^ transmission,^14^ and variant strains.^15^^,^^16^^,^^17^^,^^18^^,^^19^^,^^20^ However, recent data have shown correlations of increasing post-vaccination neutralization titers with mRNA vaccine efficacies.^21^^,^^22^ It is anticipated that antibody responses and protection from infection will wane more rapidly in older individuals,^23^^,^^24^ but there are limited and conflicting long-term data on the response and the impact of booster brands and doses.^11^^,^^17^^,^^25^^,^^26^^,^^27^^,^^28^^,^^29^^,^^30^^,^^31^^,^^32^^,^^33^ Longitudinal vaccine antibody response data outside of rigid clinical trial settings and at a time of breakthrough infection may help inform booster dose priorities and timing.^34^

With initial limited availability of vaccines against COVID-19, and in an attempt to partially immunize more Canadians, the National Advisory Committee on Immunization recommended extending the dose interval of the initial vaccine series up to 4 months and allowed vaccine brand mixing.^35^ These recommendations raised concern about vaccine efficacy especially in the elderly. Subsequently, booster doses have been made available with recommendations for administration at intervals of 6 months.

To address some of these unanswered questions, we designed the STOPCoV study- Safety and Efficacy of Preventative COVID vaccines. Our primary objective was to compare the safety and antibody responses to COVID-19 vaccines in an older community-dwelling cohort relative to a younger cohort. We hypothesized that the older group would have a less robust initial response and faster waning of antibody levels with time. We present comparative serology data to 48 weeks after the primary vaccine series (second vaccine dose). The study is ongoing to evaluate the longitudinal impact of additional booster doses, bivalent vaccines, hybrid immunity, and rate and correlates of breakthrough infections.

## Results

A total of 1,286 adults (911 older and 375 younger) were recruited between May 17– July 31, 2021. Two participants did not meet screening criteria, and 79 consenting participants did not complete any study activities, leaving 1,205 (94%) in the current analysis. Eighteen participants withdrew consent, and four participants have died; the data prior to withdrawal or death are included. Fourteen participants (all aged 30–50 years) were recruited prior to the first vaccine dose, the remaining 1,191 were recruited prior to the second vaccine dose reflecting the timelines of study initiation and vaccine distribution.

The online baseline questionnaire was completed by 1,199 (99.5%) participants. Table 1 summarizes the baseline characteristics stratified by age group. 60% of the older and 76% of the younger cohort were female. A greater proportion of the older cohort were white (93% vs.75%), more likely to be overweight and had more underlying comorbidity. Seventy-two participants (36 in each age group) reported they suspected they had COVID infection before receiving a second dose of vaccine. Fifty-one (25 in the younger cohort and 26 in the older cohort) did not have a corresponding positive test date, and so the timing of infection relative to vaccine administration could not be determined. Twenty-one participants reporting a history of COVID-19 infection reported a corresponding positive PCR for COVID; eighteen of these participants tested positive prior to a first dose of vaccine (11 aged 30–50, 7 aged ≥70), and 3, after the first dose but prior to the second dose (all aged ≥70). Of the 21 participants reporting a positive PCR, 10 also had a positive nucleocapsid protein (NP) antibody on serology testing prior to receiving a second dose. As the vaccines currently used are spike-based, reactivity to NP should only be from natural infection. Prior to the second vaccine dose, an additional 17 participants who did not report a history of COVID-19 infection (13 in the older cohort) showed a positive anti-nucleocapsid antibody indicating natural infection.

**Table 1:** Baseline participant characteristics and COVID-19 vaccine brands by age cohort

	30–50	70+
n (%)	344	861
Age^a^ (median, IQR)	41 [36, 45]	73 [71, 76]
Female or Non-Binary^a^	257 (75.6)	512 (59.6)
Racial Background		
Arab/West Indian	4 (1.2)	7 (0.8)
Black	11 (3.2)	9 (1.0)
Indigenous/Aboriginal/Indian or Native American	3 (0.9)	2 (0.2)
Latin American	7 (2.1)	0 (0.0)
South Asian	8 (2.4)	7 (0.8)
Southeast Asian	20 (5.9)	12 (1.4)
White	256 (75.3)	800 (93.1)
Other	31 (9.1)	22 (2.6)
Smoking Status^a^		
Never	241 (70.9)	434 (50.5)
Previous	68 (20.0)	390 (45.4)
Current	31 (9.1)	35 (4.1)
Comorbidities		
Diabetes	5 (1.5)	123 (14.3)
Cardiovascular Disease	17 (5.0)	414 (48.2)
Cancer	9 (2.6)	171 (19.9)
Transplant or Immunosuppressed	12 (3.5)	36 (4.2)
Chronic Obstructive Lung Disease	0 (0.0)	22 (2.6)
Asthma	48 (14.1)	76 (8.8)
Chronic Kidney Disease	3 (0.9)	17 (2.0)
Hepatitis C	2 (0.6)	3 (0.3)
Chronic Liver Disease	4 (1.2)	9 (1.0)
Chronic Blood Disease	1 (0.3)	12 (1.4)
Chronic Neurologic Disease	4 (1.2)	15 (1.7)
Dialysis	3 (0.9)	4 (0.5)
BMI^a^^,^^b^ (median, IQR)	25.68 [22.80, 29.59]	26.56 [23.68, 30.04]
BMI Category^a^^,^^b^		
Under/Healthy Weight (<25)	157 (46.9)	306 (36.1)
Overweight (2529)	97 (29.0)	328 (38.7)
Obese (≥30)	81 (24.2)	213 (25.1)
Total Number of Vaccine Doses		
1	7 (2.0)	16 (1.9)
2	89 (25.9)	96 (11.1)
3	242 (70.3)	342 (39.7)
4	6 (1.7)	407 (47.3)
Vaccine Types for First Two Doses		
Two Doses of BNT162b2	162 (48.1)	576 (68.2)
Two Doses of mRNA-1273	61 (18.1)	71 (8.4)
One Dose BNT162b2, One Dose mRNA-1273	63 (18.7)	147 (17.4)
One dose AstraZeneca Vaxzevria®, One dose BNT162b2 or mRNA-1273	38 (11.3)	30 (3.6)
Two Doses of AstraZeneca Vaxzevria®	3 (0.9)	14 (1.7)
Other Combinations or Unknown	10 (3.0)	7 (0.8)
1^st^ Booster Dose		
BNT162b2	97 (39.1)	493 (65.8)
mRNA1273	149 (60.1)	252 (33.6)
Other or Unknown	2 (0.8)	4 (0.5)
2^nd^ Booster Dose		
BNT162b2	3 (50.0)	239 (58.7)
mRNA-1273	3 (50.0)	168 (41.3)
Had ≥1 dose of mRNA-1273	210 (61.0)	415 (48.2)

BMI, body mass index; IQR, interquartile range.

a Six (0.5%) participants are missing baseline data.

b Seventeen (1.4%) participants are missing BMI data.

### Brands of initial vaccine series and boosters

All but 23 participants (1.9%) have received at least two doses of vaccine. For the initial two doses, most participants received either two BNT162b2 (Pfizer-BioNTech) or two mRNA-1273 (Moderna), with older participants more likely to receive BNT162b2 (68% vs. 48%) and younger participants more likely to receive mRNA-1273 (18% vs. 8%). 19% of the younger and 17% of the older cohort received one dose of each brand of mRNA vaccine. 11% of the younger group and 4% of the older group received one dose of AstraZeneca Vaxzevria and one dose of an mRNA vaccine. 17 participants (3 younger and 14 older cohort) received two doses of AstraZeneca Vaxevria . Overall, 997 (83%) participants received at least one booster dose by 48 weeks after the first vaccine series. For the first booster, participants 30–50 years were more likely to receive mRNA-1273 (60.1%) and those over 70 were more likely to receive BNT162b2 (65.8%). Only 6 participants in the younger cohort received a second booster dose, but almost half of the older cohort received a second booster, with 239 (59%) of these receiving BNT162b2 and 168 (41%) receiving mRNA-1273. For booster doses of mRNA-1273, younger participants received a dosage of 50 μg while older participants received a dosage of 100 μg as per public health guidelines.

### Safety

The 7-day electronic diaries were completed for 49 (4%) participants after first vaccine dose, 957 (79%) after second vaccine dose, and 756 (63%) after the first booster. After the first dose, the most commonly reported adverse events were pain near the injection site (63%), fatigue (53%), and malaise (39%). The most commonly reported adverse events after the second vaccine dose (Table 2) were pain near the injection site (88.5%), fatigue (68.5%), muscle aches or pains (53.6%), malaise (50.6%), and headaches (45.2%). Younger participants were more likely to report each adverse event and more likely to experience at least one event to a moderate (some interference with activity) or severe (significant, prevents daily activity) degree (82%) compared to the older participants (39%, p < 0.0001). Adverse events after the second dose were more likely for participants receiving mRNA-1273 compared to BNT162b2 (Table 2). 49 (5%) participants reported no adverse events to the second vaccine dose. At seven days after the second dose, 105 (11.6%) of participants reported fatigue, but all other adverse symptoms were reported by ≤ 5% of participants. The type and frequency of adverse events reported after the first booster dose (Table 3) were similar to those of the second dose; 85% reported pain, 65%, fatigue, 49%, muscle aches, 49%, malaise, and 44%, headaches. All were more commonly reported by those receiving mRNA-1273 compared to BNT162b2. At each of 12 monthly follow-ups, 1.5%–4.3% of participants reported persistent adverse events thought to be vaccine related.

**Table 2:** Second dose adverse vaccine symptoms by brand and age

	30–50			70+		
	BNT162b2	mRNA-1273	p	BNT162b2	mRNA-1273	p^a^
n (%)^b^	177	147		608	218	
Completed ≥1 diary	140 (79.1)	122 (83.0)	0.46	498 (81.9)	180 (82.6)	0.91
Redness near injection site	18 (12.9)	31 (25.4)	0.015	52 (10.4)	30 (16.7)	0.039
Swelling near injection site	39 (27.9)	40 (32.8)	0.46	61 (12.2)	51 (28.3)	<0.0001
Hives (not at injection site)	12 (8.6)	13 (10.7)	0.72	27 (5.4)	11 (6.1)	0.88
Pain near injection site	136 (97.1)	118 (96.7)	0.99	421 (84.5)	162 (90.0)	0.092
Any malaise	86 (61.4)	108 (88.5)	<0.0001	188 (37.8)	92 (51.1)	0.0024
Any headaches	88 (62.9)	99 (81.1)	0.0018	162 (32.5)	72 (40.0)	0.086
Any fatigue	112 (80.0)	115 (94.3)	0.0014	297 (59.6)	120 (66.7)	0.12
Any chills	41 (29.3)	76 (62.3)	<0.0001	34 (6.8)	42 (23.3)	<0.0001
Any muscle aches or pains	80 (57.1)	97 (79.5)	0.00020	221 (44.4)	107 (59.4)	0.00073
Any joint aches or pains	42 (30.0)	68 (55.7)	<0.0001	128 (25.7)	70 (38.9)	0.0012
Any nausea	42 (30.0)	63 (51.6)	0.00058	68 (13.7)	42 (23.3)	0.0037
Any fevers	17 (12.1)	51 (41.8)	<0.0001	17 (3.4)	19 (10.6)	0.00052
Other serious problem related to the vaccine	17 (12.1)	34 (27.9)	0.0023	36 (7.2)	27 (15.0)	0.0034
≥1 moderate or severe symptom	104 (74.3)	113 (92.6)	0.00017	170 (34.1)	95 (52.8)	<0.0001

a p values are from chi-square tests.

b The n (%) are the proportions of those completing the diary who reported each of the symptoms.

**Table 3:** Third dose adverse vaccine symptoms by brand and age

	30–50			70+		
	BNT162b2	mRNA-1273	p	BNT162b2	mRNA-1273	p^a^
n (%)^b^	97	149		493	252	
Completed ≥1 diary	79 (81.4)	121 (81.2)	0.99	366 (74.2)	187 (74.2)	0.99
Redness near injection site	11 (13.9)	25 (20.7)	0.31	48 (13.1)	45 (24.1)	0.0017
Swelling near injection site	13 (16.5)	41 (33.9)	0.011	48 (13.1)	57 (30.5)	<0.0001
Hives (not at injection site)	2 (2.5)	9 (7.4)	0.24	21 (5.7)	12 (6.4)	0.90
Pain near injection site	76 (96.2)	117 (96.7)	0.99	280 (76.5)	169 (90.4)	0.00013
Any malaise	47 (59.5)	92 (76.0)	0.020	130 (35.5)	99 (52.9)	0.00012
Any headaches	55 (69.6)	78 (64.5)	0.55	110 (30.1)	88 (47.1)	0.00012
Any fatigue	64 (81.0)	103 (85.1)	0.57	201 (54.9)	118 (63.1)	0.080
Any chills	18 (22.8)	42 (34.7)	0.10	60 (16.4)	55 (29.4)	0.00054
Any muscle aches or pains	44 (55.7)	79 (65.3)	0.22	144 (39.3)	103 (55.1)	0.00060
Any joint aches or pains	23 (29.1)	43 (35.5)	0.43	76 (20.8)	70 (37.4)	<0.0001
Any nausea	21 (26.6)	36 (29.8)	0.75	40 (10.9)	31 (16.6)	0.081
Any fevers	8 (10.1)	16 (13.2)	0.66	15 (4.1)	15 (8.0)	0.084
Other serious problem related to the vaccine	17 (21.5)	15 (12.4)	0.13	23 (6.3)	17 (9.1)	0.30
≥1 moderate or severe symptom	42 (53.2)	91 (75.2)	0.0021	110 (30.1)	96 (51.3)	<0.0001

a p values are from chi-square test.

b The n (%) are the proportions of those completing the diary who reported each of the symptoms.

### Serology results

Antibody results are available for 14 (1%) participants prior to their first vaccine, 84 (7%) three weeks after first vaccine, 969 (80%) prior to second vaccine, and 1006 (84%) two weeks, 940 (78%) 12 weeks, 927 (77%) 24 weeks, and 877 (73%) 48 weeks after the second dose.

Results were obtained as relative ratios to a synthetic standard included as a calibration curve on each assay plate.^36^ All values were calibrated to the World Health Organization (WHO) international standard enabling comparisons to other datasets and are presented as binding antibody units (BAU)/mL. Seropositivity cutoffs were 11.28 BAU/mL for anti-spike immunoglobulin G (IgG), 30.97 BAU/mL for anti-RBD (receptor-binding domain) IgG, and 34.46 BAU/mL for NP. Specimen overall positivity to vaccine required the IgG to both spike and RBD to be above threshold.

**Figure 1:**
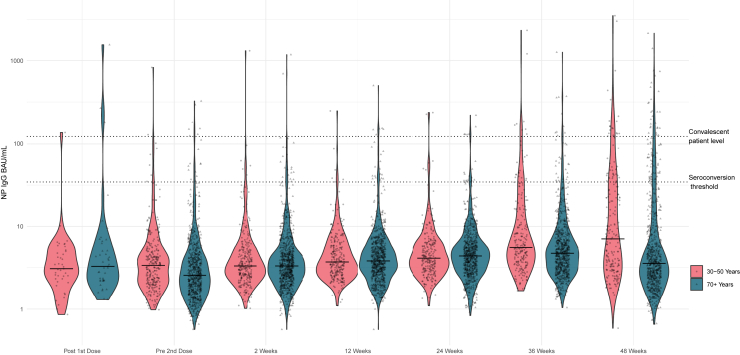
Violin plot of nucleoprotein IgG BAU/ml by time since vaccine dose and by age cohort

**Figure 2:**
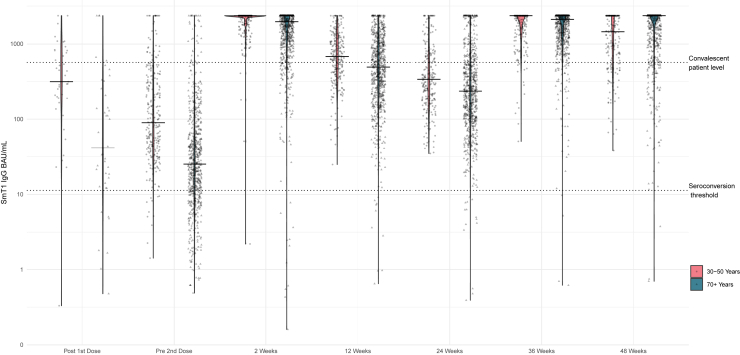
Violin plot of spike IgG BAU/mL by time since vaccine dose and by age cohort

**Figure 3:**
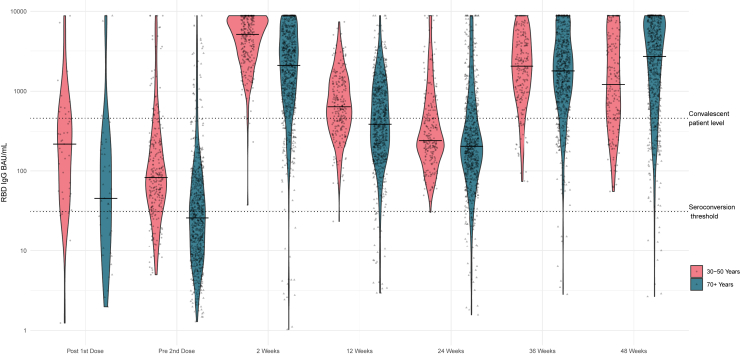
Violin plot of receptor binding domain IgG BAU/mL by time since vaccine dose and by age cohort

Figure 1, Figure 2, Figure 3 display the violin plots of anti- NP, anti-spike, and anti-RBD in BAU/mL by time and age cohort.

Detailed antibody levels by time and age are provided in Table 4. As not all participants submitted samples at each time point, the denominators and % are based on the numbers who did submit at each sampling time. In the older cohort, the proportion with overall positive serology increased from 320 (45%) to 726 (98.5%) after the second vaccine dose. There was a corresponding increase in median (interquartile range [IQR]) anti-RBD levels from 26 (10, 68) BAU/mL before the second vaccine dose to 2096 (837, 4726) BAU/mL two weeks after the second dose. At 12 weeks after second vaccine dose, the percentage with overall positive serology remained high (96.3%), but the median RBD value decreased to 387 (187, 975) BAU/mL. At 24 weeks after second vaccine dose, the percentage with overall positive serology decreased to 93% and the median antibody level declined further to 206 (104, 514) BAU. Median anti-RBD IgG increased to 1788 (810, 4034) BAU/mL at 36 weeks and reached the highest levels at 48 weeks (2705 (785, 6464) BAU/mL) reflecting a response to booster doses. At 36 weeks, 655 (95%) of the cohort submitting dried blood spots (DBS) had received one booster and 12 (1.7%) had received two boosters. By 48 weeks, 289 (43%) had received one booster and 365 (54%) had received two boosters. The presence of moderate-to-severe vaccine reactogenicity was associated with higher anti-RBD levels at 2, 12, and 24 weeks after the second vaccine (Table 5).

**Table 4:** Median (IQR) antibody (IgG BAU/ml) to nucleocapsid proteins (NP), spike protein, and receptor-binding domain (RBD) by time since vaccination dose by age cohort

	30–50 years	70 + years	p^a^
N	344	861	
3 Weeks Post 1^st^ Dose			
n (%) with a result	41 (11.9)	43 (5.0)	<0.0001
NP			
Median [IQR] BAU/mL	3.08 [2.33, 4.98]	3.29 [2.24, 5.64]	0.48
n (%) Positive	1 (2.4)	3 (7.0)	0.62
Spike			
Median [IQR] BAU/mL	313 [189, 805]	41 [15, 230]	<0.0001
n (%) Positive	40 (97.6)	36 (83.7)	0.058
RBD			
Median [IQR] BAU/mL	219 [54, 516]	45 [16, 187]	0.0012
n (%) Positive	35 (85.4)	26 (60.5)	0.014
Spike AND RBD positive^b^	35 (85.4)	26 (60.5)	0.014
Pre 2^nd^ Dose			
n (%) with a result	262 (76.2)	707 (82.1)	0.020
NP			
Median [IQR] BAU/mL	3.38 [2.13, 5.11]	2.57 [1.67, 4.23]	<0.0001
n (%) Positive	8 (3.1)	18 (2.5)	0.66
Spike			
Median [IQR] BAU/mL	90 [40, 307]	25 [11, 65]	<0.0001
n (%) Positive	251 (95.8)	519 (73.4)	<0.0001
RBD			
Median [IQR] BAU/mL	83 [40, 216]	26 [10, 68]	<0.0001
n (%) Positive	218 (83.2)	321 (45.4)	<0.0001
Spike AND RBD positive^b^	218 (83.2)	320 (45.3)	<0.0001
2 Weeks Post 2^nd^ Dose			
n (%) with a result	269 (78.2)	737 (85.6)	0.0026
NP			
Median [IQR] BAU/mL	3.32 [2.48, 5.19]	3.32 [2.33, 4.94]	0.38
n (%) Positive	6 (2.2)	23 (3.1)	0.53
Spike			
Median [IQR] BAU/mL	2359 [2094, 2359]	1966 [1065, 2359]	<0.0001
n (%) Positive	268 (99.6)	726 (98.5)	0.20
RBD			
Median [IQR] BAU/mL	5180 [2690, 8067]	2096 [837, 4726]	<0.0001
n (%) Positive	269 (100)	722 (98.0)	0.015
Spike AND RBD positive^b^	268 (99.6)	722 (98.0)	0.084
12 Weeks Post 2^nd^ Dose			
# (%) with a result	241 (70.1)	699 (81.2)	<0.0001
NP			
Median [IQR] BAU/mL	3.71 [2.73, 5.83]	3.82 [2.66, 5.74]	0.90
n (%) Positive	6 (2.5)	22 (3.1)	0.83
Spike			
Median [IQR] BAU/mL	670 [394, 1177]	488 [218, 1025]	<0.0001
n (%) Positive	241 (100)	689 (98.6)	0.073
RBD			
Median [IQR] BAU/mL	637 [360, 1295]	387 [187, 975]	<0.0001
n (%) Positive	240 (99.6)	673 (96.3)	0.059
Spike AND RBD positive^b^	240 (99.6)	673 (96.3)	0.059
24 Weeks Post 2^nd^ Dose			
# (%) with a result	231 (67.2)	696 (80.8)	<0.0001
NP			
Median [IQR] BAU/mL	4.12 [2.96, 6.26]	4.38 [2.98, 6.32]	0.69
n (%) Positive	6 (2.6)	19 (2.7)	0.99
Spike			
Median [IQR] BAU/mL	337 [181, 728]	235 [103, 570]	<0.0001
n (%) Positive	231 (100)	682 (98.0)	0.027
RBD			
Median [IQR] BAU/mL	242 [162, 588]	206 [106, 514]	0.00036
n (%) Positive	230 (99.6)	650 (93.4)	<0.0001
Spike AND RBD positive^b^	230 (99.6)	650 (93.4)	<0.0001
36 Weeks Post 2^nd^ Dose			
n (%) with a result	215 (62.5)	690 (80.1)	<0.0001
NP			
Median [IQR] BAU/mL	5.53 [3.69, 13.4]	4.72 [3.08, 7.64]	<0.0001
n(%) Positive	25 (11.6)	32 (4.6)	0.00058
Spike			
Median [IQR] BAU/mL	2359 [1399, 2359]	2110 [1227, 2359]	0.075
n (%) Positive	215 (100)	686 (99.4)	0.58
RBD			
Median [IQR] BAU/mL	2048 [966, 4193]	1788 [810, 4034]	0.19
n (%) Positive	215 (100)	682 (98.8)	0.21
Spike AND RBD positive^b^	215 (100)	682 (98.8)	0.21
48 Weeks Post 2^nd^ Dose			
n (%) with a result	200 (58.1)	677 (78.6)	<0.0001
NP			
Median [IQR] BAU/mL	7.07 [3.28, 30.25]	3.57 [2.30, 7.75]	<0.0001
n (%) Positive	47 (23.5)	80 (11.8)	<0.0001
Spike			
Median [IQR] BAU/mL	1450 [598, 2359]	2359 [1162, 2359]	<0.0001
n (%) Positive	200 (100)	674 (99.6)	0.99
RBD			
Median [IQR] BAU/mL	1211 [461, 3917]	2705 [785, 6464]	<0.0001
n(%) Positive	200 (100)	667 (98.5)	0.13
Spike AND RBD positive^b^	200 (100)	667 (98.5)	0.13

IQR, interquartile range; RBD, receptor-binding domain; BAU, binding antibody units; NP, nucleocapsid protein.

a p values are from Kruskal-Wallis tests for median BAU/mL values and Fisher’s Exact tests for n(%) Positive values.

b Overall seropositivity is defined as antibody positive to both spike and RBD.

**Table 5:** Median [IQR antibody (IgG BAU/ml) to receptor-binding domain (RBD) by self-reported reactogenicity to second vaccine dose

	Mild or No reactogenicity	Moderate to severe reactogenicity	P^a^
30–50 Cohort			
N	47	220	
2 Weeks Post 2^nd^ Dose	4927 [1841, 6167]	5298 [2996, 8305]	0.11
12 Weeks Post 2^nd^ Dose	485 [233, 857]	712 [406, 1376]	0.0054
24 Weeks Post 2^nd^ Dose	196 [109, 241]	278 [182, 633]	0.0064
48 Weeks Post 2^nd^ Dose	1033 [474, 3830]	1250 [465, 3941]	0.91
70+ Cohort			
N	421	269	
2 Weeks Post 2^nd^ Dose	1834 [757, 3635]	2874 [1156, 5660]	0.00012
12 Weeks Post 2^nd^ Dose	342 [162, 832]	545 [251, 1134]	<0.0001
24 Weeks Post 2^nd^ Dose	177 [92, 415]	250 [136, 689]	<0.0001
48 Weeks Post 2^nd^ Dose	2484 [840, 6658]	2674 [753, 5916]	0.84

IQR, interquartile range; RBD, receptor-binding domain; BAU, binding antibody units.

a p values are from Kruskal-Wallis tests.

Compared to the 991 participants who seroconverted, the 15 (2%) participants negative for anti-RBD IgG two weeks after the second dose were older (100% vs. 73%, p = 0.015), white (100% vs. 88%, p = 0.24), women (67% vs. 65%, p = 0.99), prior smokers (67% vs. 38%, p = 0.039), obese (60% vs. 24%, p = 0.0059), had underlying cardiovascular disease (60% vs. 36%, p = 0.059), cancer (40% vs. 16%, p = 0.023), chronic blood disease (20% vs. 1%, p = 0.00071), transplant or immunosuppression (40% vs. 3%, p < 0.0001), and received two doses of BNT162b2 (73% vs. 63%, p = 0.067). Ten of these participants eventually developed positive antibody. One developed spike and RBD antibody by 12 weeks with 2 doses. Six developed spike antibody with 2 doses (four at 2 weeks and 2 at 12 weeks) and later developed RBD at 36 weeks after 1 (n = 5) or 2 (n = 1) boosters. One developed spike antibodies at 24 weeks after a first booster and RBD antibodies at 36 weeks after a second booster. Two participants developed both spike and RBD antibodies at 36 weeks after one booster dose. Five participants never developed antibody to either spike or RBD. One had 1 subsequent negative result at 12 weeks, and one had 2 subsequent negative results at 12 and 24 weeks with no boosters reported. The remaining three had at least 3 subsequent negative results and were all negative at 48 weeks despite receiving one (n = 2) or two (n = 1) boosters. Of the 5 participants that never developed antibody, 4 were female, all were overweight or obese, 4 were immune suppressed and taking prescription medication for it, 4 had 3 or 4 doses of BNT162b2, and 1 had 2 doses of AstraZeneca Vaxzevria.

The proportion of younger participants with overall positive serology increased from 83% to 100% before the second vaccine dose to two weeks after the second dose. Younger participants demonstrated an increase in median (IQR) antibody from 83 (40, 215) BAU/mL before the second vaccine dose to 5180 (2690, 8067) BAU/mL two weeks after the second dose. At 12 and 24 weeks after the second dose, 99.6% had overall positive serology, but the median RBD decreased to 637 (360, 1295) BAU/mL at 12 weeks and 242 (162, 588) BAU/mL at 24 weeks (Figure 3). At 36 weeks, 192 (89%) of the cohort submitting DBS samples had received one booster and 2 (1%) had received two. At 48 weeks 181 (91%) had received one booster and 6 (3%) had received two. At 48 weeks all of the younger participants had overall positive serology, and the median anti-RBD level increased at 48 weeks to 1211 (461, 3917) BAU/mL. Among younger participants, those with moderate-to-severe vaccine reactogenicity had similar levels of anti-RBD compared to those with mild or no reactogenicity at 2 and 48 weeks after the second vaccine but had higher levels of anti-RBD at 12 and 24 weeks (Table 5).

For both the older and the younger cohort, the trends in anti-spike antibody were similar to those for anti-RBD (Table 4, Figure 2). For the younger cohort at 2 and 36 weeks and the older at 48 weeks, the median value for anti-spike antibody is capped at the limit of the upper range of the assay, and hence the values are underestimated.

Table 6 demonstrates antibody results by time and vaccine brand. For both the younger (p < 0.01) and older cohort (p < 0.0001), at 2 and 24 weeks after second dose, RBD antibody levels were higher for those receiving two doses of mRNA-1273 or one dose of mRNA-1273 and one dose of BNT162b2 and lower for those receiving two doses of BNT162b2. RBD antibody levels at 48 weeks did not differ by original vaccine brand in either age cohort.

**Table 6:** Median [IQR antibody (IgG BAU/ml) to receptor-binding domain (RBD) by brand and age category with Time

	Two doses of BNT162b2	Two doses of mRNA-1273	One dose BNT162b2, One dose mRNA-1273	One dose AstraZeneca Vaxzevria®, one dose BNT162b2 or mRNA-1273	Other combinations or unknown	p^a^
30–50 Cohort						
n	162	61	63	38	13	
2 Weeks Post 2^nd^ Dose						
n (%) with a result	131 (80.9)	49 (80.3)	53 (84.1)	32 (84.2)	3 (23.1)	<0.0001
Median [IQR] BAU/mL	3983 [2331, 7170]	6030 [4399, 7857]	6810 [4417, 8829]	4261 [3122, 6678]	1278 [864, 3623]	0.00027
# (%) Positive	131 (100)	49 (100)	53 (100)	32 (100)	3 (100)	n/a
24 Weeks Post 2^nd^ Dose						
n (%) with a result	108 (66.7)	41 (67.2)	51 (81.0)	29 (76.3)	2 (15.4)	0.00013
Median [IQR] BAU/mL	227 [133, 499]	425 [224, 771]	263 [192, 637]	204 [127, 354]	3244 [1740, 4747]	0.010
# (%) Positive	107 (99.1)	41 (100)	51 (100)	29 (100)	2 (100)	0.89
48 Weeks Post 2^nd^ Dose						
n (%) with a result	97 (59.9)	36 (59.0)	40 (63.5)	24 (63.2)	3 (23.1)	0.10
Median [IQR] BAU/mL	886 [403, 3382]	2432 [630, 4557]	1211 [434, 3886]	1870 [573, 7751]	748 [523, 2015]	0.20
n(%) Positive	97 (100)	36 (100)	40 (100)	24 (100)	3 (100)	n/a
70+ Cohort						
n	576	71	147	30	21	
2 Weeks Post 2^nd^ Dose						
n (%) with a result	499 (86.6)	63 (88.7)	132 (89.8)	27 (90.0)	16 (76.2)	0.445
Median [IQR] BAU/mL	1718 [683, 3528]	5427 [2940, 8829]	3407 [1586, 6582]	1775 [904, 5196]	462 [99, 1123]	<0.0001
# (%) Positive	488 (97.8)	62 (98.4)	131 (99.2)	27 (100)	14 (87.5)	0.032
24 Weeks Post 2^nd^ Dose						
n (%) with a result	483 (83.9)	55 (77.5)	120 (81.6)	23 (76.7)	15 (71.4)	0.34
Median [IQR] BAU/mL	185 [93, 447]	451 [237, 1134]	227 [135, 546]	135 [96, 283]	119 [26, 1070]	<0.0001
n (%) Positive	449 (93.0)	55 (100)	115 (95.8)	21 (91.3)	10 (66.7)	0.00014
48 Weeks Post 2^nd^ Dose						
n (%) with a result	469 (81.4)	54 (76.1)	114 (77.6)	24 (80.0)	16 (76.2)	0.71
Median [IQR] BAU/mL	2444 [689, 5999]	3618 [1448, 8706]	3213 [952, 6939]	3396 [1518, 6536]	2698 [1225, 5130]	0.13
n (%) Positive	460 (98.1)	53 (98.1)	114 (100)	24 (100)	16 (100)	0.56

IQR, interquartile range; n/a, not applicable; BAU, binding antibody units.

a p values are from Kruskal-Wallis tests for median BAU/mL values and chi-square tests for n(%) Positive values.

Anti-RBD response to a single dose of vaccine (prior to the second dose) in BAU/mL is modeled using median regression in Table 7. All but six samples were in the linear range of the assay. In univariable models, older age, male sex, and all included comorbidities were associated with lower levels of RBD antibody while having a first dose of mRNA-1273 (compared to other vaccine brands) was associated with higher antibodies. In the multivariable model, older age, male sex, cancer, and diabetes remained associated with lower antibodies and mRNA-1273 corresponded to higher antibodies.

**Table 7:** Median regression model of RBD (receptor-binding domain) IgG BAU/ml prior to second dose

	Univariable		Multivariable	
	β (95% CI)	p	β (95% CI)	p
Age 70+	−56.8 (−65, −47)	<0.0001	−44.2 (−53.8, −32.7)	<0.0001
Female or non-binary	14.8 (8.08, 21.9)	0.00053	5.76 (1.42, 11)	0.044
Cardiovascular Disease	−26.3 (−31, −18.4)	<0.0001	−4.16 (−8.84, 2.92)	0.19
Cancer	−19.3 (−27.2, −14.3)	<0.0001	−9.2 (−13.1, −3.67)	0.00062
Diabetes	−21.6 (−27.4, −15.7)	<0.0001	−6.53 (−9.94, −2.08)	0.048
Transplant or Immunosuppressed	−18.4 (−29, −6.75)	0.0030	−9.42 (−19.1, 0.017)	0.057
BMI (per +10)	−4.87 (−8.59, 1.29)	0.17	0.32 (−4.37, 3.36)	0.92
Ever had positive COVID test	49.6 (21.7, 1454)	0.54	44.8 (1.37, 1661)	0.16
1^st^ Dose was mRNA-1273	127 (95.9, 179)	<0.0001	117 (78.3, 158)	<0.0001
White	−26.3 (−41.4, −16.5)	0.00032	n/a	

BMI, body mass index; CI, confidence interval; n/a, not applicable; RBD, receptor-binding domain.

Table 8 is the univariable and multivariable median regression models of anti-RBD in BAU/mL at 2 weeks after second vaccine dose. 890 (88%, overall, 82% for younger participants and 91% for older participants) samples were in the linear range of the assay in BAU/ml. After adjusting for covariates, older participants had lower RBD antibody levels two weeks after the second dose compared to younger participants (p < 0.0001). Male sex (p = 0.013), a cancer diagnosis (p = 0.0094), and lower body mass index (BMI) (p = 0.00011) were associated with lower anti-RBD. Receiving one or two doses of mRNA-1273 was associated with higher levels of anti-RBD IgG.

**Table 8:** Median regression model of RBD (receptor-binding domain) IgG BAU/ml at 2 weeks after second dose

	Univariable		Multivariable	
	β (95% CI)	p	β (95% CI)	p
Age 70+	−3084 (323, −955)	<0.0001	−1914 (−2581, −1308)	<0.0001
Female or non-binary	965 (296, 326)	0.0011	507 (225, 867)	0.013
Cardiovascular Disease	−1115 (285, −391)	0.0001	−280 (−632, 120)	0.19
Cancer	−1053 (334, −315)	0.0017	−495 (−837, −193)	0.0094
Diabetes	−1153 (390, −296)	0.0032	−258 (−758, 359)	0.52
Transplant or Immunosuppressed	−1112 (755, −147)	0.14	−582 (−1289, 92.6)	0.16
BMI (per +10)	531 (168, 971)	0.063	853 (368, 1067)	0.00011
Ever had positive COVID test	1620 (1788, 90.6)	0.37	1507 (192, 2710)	0.070
Any mRNA-1273 Doses	3117 (317, 985)	<0.0001	2225 (1613, 2762)	<0.0001
White	−1514 (508, −298)	0.0029	n/a	

BMI, body mass index; CI, confidence interval; n/a, not applicable; RBD, receptor-binding domain.

Median regression models of anti-RBD at 24 weeks after the second vaccine dose are presented in Table 9. At 24 weeks, 910 (98% overall and for both age cohorts) samples were in the linear range of the assay. Anti-RBD levels at this time point were not different by age cohort in either univariable or multivariable models. Female or non-binary participants (p = 0.016) as well as those with a booster dose (p = 0.00031) or one or more doses of mRNA-1273 (p = 0.0002) had higher anti-RBD ratios. Cancer and BMI were not associated with anti-RBD levels at 24-week after second vaccine dose.

**Table 9:** Median regression model of RBD (receptor-binding domain) IgG BAU/ml at 24 weeks after second dose

	Univariable		Multivariable	
	β (95% CI)	p	β (95% CI)	p
Age 70+	−35.4 (−85.4, −16.6)	0.163	−11.3 (−50.4, 16.3)	0.59
Female or non-binary	55.6 (25.7, 77.5)	0.0013	37.5 (4.24, 60.2)	0.016
Cardiovascular Disease	−45.7 (−82.3, −15.8)	0.017	−23 (−52.6, 2.27)	0.14
Cancer	−27.6 (−54, 2.28)	0.13	−8.33 (−34.4, 16.1)	0.51
Diabetes	−71.4 (−110, −7.86)	0.016	−32.9 (−67.8, −1.75)	0.13
Transplant or Immunosuppressed	5.7 (−99.7, 121)	0.94	−94.6 (−237, 54.9)	0.36
BMI (per +10)	−7.81 (−26.6, 11.3)	0.64	−21.8 (−49.5, 18.7)	0.15
Ever had positive COVID test	105 (0.31, 242)	0.17	84.1 (27.7, 296)	0.37
Had a booster	1594 (818, 2211)	0.00028	1600 (953, 2239)	0.00031
Any mRNA-1273 Doses	102 (63, 149)	<0.0001	87 (49.9, 127)	0.0002
White	23.7 (1.19, 44.2)	0.17	n/a	

BMI, body mass index; CI, confidence interval; n/a, not applicable; RBD, receptor-binding domain.

Median regression models of anti-RBD at 48 weeks are presented in Table 10; 754 (86% overall, 91% for the younger cohort, 85% for the younger cohort) samples were in the linear range of the assay. At 48 weeks after the initial vaccine series, having had a positive COVID test (p < 0.0001) and receipt of any dose of mRNA-1273 (p < 0.0001) were associated with higher levels of anti-RBD (p < 0.0001). At this time point, almost all participants in the 30–50 age group had received 3 total doses of vaccine and almost half of participants over 70 had 4 total doses. Older participants with 3 doses had similar antibody levels to younger participants with 3 doses in a univariable model but showed a higher level of anti-RBD after adjusting for covariates. This apparent age differential may be confounded by the different dosages of mRNA-1273 administered to different age groups as boosters. In a sensitivity analysis where participants receiving mRNA-1273 as their first booster dose (149 younger participants and 252 older participants who were demographically and clinically similar to participants receiving other types of boosters within their cohorts) were excluded, older participants with 3 doses were not different from younger participants with 3 doses after adjusting for covariates (β (95% CI) = 199 (−133, 421), p = 0.45). All of the regression models remained robust when mean anti-RBD antibody levels were used instead of medians.

**Table 10:** Median regression model of RBD (receptor-binding domain) IgG BAU/ml at 48 weeks after second dose

	Univariable		Multivariable	
	β (95% CI)	p	β (95% CI)	p
Age & Dose				
30–50 with 3 Doses	Ref.		Ref.	
70+ with 3 Doses	−133 (−554, 513)	0.65	451 (214, 747)	0.041
70+ with 4 Doses	2726 (2195, 3250)	<0.0001	2674 (2415, 3116)	<0.0001
Female or non-binary	−392 (−1346, 307)	0.27	93.2 (−91.2, 358)	0.57
Cardiovascular Disease	987 (212, 1510)	0.0043	40.9 (−218, 271)	0.84
Cancer	1223 (454, 1900)	0.014	161 (−87.4, 572)	0.47
Diabetes	−71.8 (−1163, 811)	0.91	−324 (−547, −89.3)	0.11
Transplant or Immunosuppressed	−1706 (−2189, −1039)	<0.0001	−916 (−2599, −538)	0.097
BMI (per +10)	521 (−205, 1306)	0.22	266 (67, 521)	0.097
Ever had positive COVID test	4981 (4119, 5936)	<0.0001	4871 (3786, 5166)	<0.0001
Any mRNA-1273 Doses	1509 (737, 2008)	<0.0001	888 (582, 1163)	<0.0001
White	928 (479, 1625)	0.0066	n/a	

BMI, body mass index; CI, confidence interval; n/a, not applicable; RBD, receptor-binding domain.

### Breakthrough COVID infections following initial vaccine series

Two weeks after the second vaccine dose, breakthrough infection was observed in 9 participants (during Alpha/Gamma wave) as demonstrated by the development of positive anti-NP antibodies. At 12 and 24 weeks (Delta wave) after the second dose, an additional 12 and 17 participants respectively had breakthrough infection identified by either a positive anti-nucleocapsid or self-report of a positive PCR or rapid antigen test (RAT). A further increase in the rate of breakthrough infections began at week 36 (Omicron BA.1/BA.2 waves) where 63 new participants recorded a positive COVID test (32 of which also had a positive anti-nucleocapsid). By 48 weeks (Omicron BA.5 wave) an additional 130 participants reported a positive COVID test of whom 82 developed a positive anti-nucleocapsid. Overall, of 1167 participants that did not have a positive COVID test or positive antibody to NP prior to receiving their second dose of vaccine, 231 (20%) including 95/329 (29%) of the younger and 136/838 (16%, p < 0.0001) of the older cohort developed breakthrough infection to 48 weeks after the second vaccine dose. No individual with a breakthrough infection required hospitalization or died.

## Discussion

We report the real-time prospective longitudinal antibody response in the largest cohort of community-dwelling elders relative to a younger community cohort. We obtained our serology values as normalized ratios to NP, spike, and RBD and have converted our antibody values to the WHO standard BAU/ml allowing comparison between studies. Our older cohort (≥70 years of age) had a less robust antibody response to the initial COVID-19 vaccine series than the younger cohort (aged 30–50 years), but this difference diminished with booster doses. Only 83% of the younger cohort and 45% of the older cohort had overall positive serology (IgG to both RBD and spike above threshold) prior to the second dose (p <0 .0001). Prior to the second dose of the initial vaccine series, lower antibody levels were seen in the older cohort, male gender, and those with underlying comorbidity such as cancer, diabetes, and immunosuppression, whereas levels were higher in those who received mRNA-1273. Overall seropositivity increased to 98% of older and 100% of younger participants after the second of the initial COVID vaccine doses (p = 0.084). Immunity waned such that at 24 weeks 99.6% of the younger but only 93.4% of the older cohort had a positive response. However, this increased to 100%/98.5% in the younger and older cohorts, respectively, at 48 weeks demonstrating the positive impact of booster doses. Although successful in partially immunizing more persons with a single dose in a situation of limited supply, our data reinforce the importance of the two-dose vaccination series and point to the need for booster doses especially in the elderly.^37^ As noted by others, there are a minority of older individuals (2% in our series without positive serology after vaccine) that might require earlier or more frequent booster dosing. Although the numbers are small, there were certain characteristics including obesity, underlying comorbidity, and vaccine brand (two doses BNT162b2) of those who did not seroconvert after the initial series. In our cohort all but 5 participants were able to develop antibody after booster dosing.

Antibody to RBD is thought to most closely reflect neutralizing antibody^15^^,^^38^ at the population level. In our study, the median RBD BAU/mL at two weeks after the initial series was higher in the younger than that in the older cohort with the peak values in both age groups being higher than the median values observed in convalescent sera of those with prior COVID infection. At weeks 12 and 24 after the initial series, there was a significant decline in median RBD BAU/mL in both cohorts, but levels remained higher for the younger cohort. By weeks 36 and 48, the values increased in response to booster dosing, and now the older cohort had higher median BAU/mL than the younger cohort. It is to be noted that the dose of mRNA-1273 in the booster dose was higher for the older than that for the younger cohort which may contribute to this finding. Indeed, in our sensitivity analysis excluding participants with an mRNA-1273 booster, the antibody levels in the two cohorts are comparable. We observed a greater heterogeneity in the antibody response in the older cohort relative to the younger cohort especially with time. It is unclear whether those with lower responses have less protection and could benefit from different vaccine booster schedules. Nonetheless, the antibody responses especially to booster doses are robust.

There was initially considerable public concern regarding vaccine brand and brand mixing. In our study at two weeks after initial series those who received two doses of mRNA-1273 or one dose of mRNA-1273 and one dose of BNT162b2 had higher RBD antibody levels than those receiving two doses of BNT162b2. This is consistent with others who have demonstrated comparable antibody responses with brand mixing^33^^,^^39^^,^^40^ and higher antibody levels with mRNA-1273.^41^^,^^42^^,^^43^ In multivariable models, having one or more doses of mRNA-1273 corresponded to higher RBD antibody levels at 2, 24, and 48 weeks after the initial vaccine series.

In other infection/vaccine studies such as influenza,^44^ older adults and compromised persons have a less robust response, likely a consequence of immunosenescence, leading to changes in vaccine strategies. This may also be necessary with COVID-19 vaccines.^32^ In our study, in addition to age, male sex, lower weight, and prior cancer were associated with lower peak antibody response to the initial series. This is consistent with data from other studies which have shown lower antibody^45^ and neutralizing antibody responses and faster antibody level declines in older adults^27^^,^^45^ and adults in long-term care^41^ compared to health-care staff.^46^ A United Kingdom modeling study also predicted a lower vaccine response in older participants and those with long-term health conditions.^13^ Consistent with these observations, vaccine effectiveness against COVID-19 hospitalization declined over time in a study of persons >65 years.^47^

In our multivariate models, although comorbidity and age impacted the response to the initial vaccine series, the effect of these covariates was not significant after 48 weeks, suggesting that booster dosing can overcome the impact of age, gender, and comorbidity on the initial antibody response. We will continue to follow our cohort to determine if this will translate into similar rates of protection from breakthrough infection and disease severity.

Understanding whether a specific antibody level has predictive power for vaccine efficacy at the individual level and what threshold confers a desired level of protection in different populations will help inform timing of further doses.^10^^,^^48^ In our cohort, overall, 231 (20%) had a breakthrough infection, with most infections occurring during the Omicron BA.5 wave of infection. Our observed breakthrough rate is much lower than that modeled for the overall Canadian population where the infection-acquired seroprevalence increased significantly between August 2021 and September 30, 2022: from 4.9% (95% credible interval [CrI]: 3.7 to 5.9) in the pre-Delta wave to 67.5% (95% CrI: 64.3 to 70.7) by the end of September 2022—after nine months with circulating Omicron variants.^49^^,^^50^ Other studies have demonstrated the rates were lower in the older age group but were still higher than we observed. Although our lab^51^ and others^52^^,^^53^ have demonstrated similar results when comparing plasma to DBS, use of different antibody assays or cutoff values for positivity impairs the ability to compare results. The lower rates in our cohort may result from a combination of strong adherence to vaccine and booster schedules or better adherence to public health advice on social distancing. As seen in other series,^54^ none of our participants required hospitalization or died as a result of their breakthrough COVID infection.

In our study, the vaccines were safe and well tolerated. Although 95% participants reported at least one adverse event, these tended to be mild to moderate and short lived. Only 14% reported at least one symptom in the severe category. Adverse events were reported more commonly in our younger cohort and from those receiving mRNA-1273. Despite receiving a lower dose of mRNA-1273 for a booster in the younger cohort, the number of reported adverse events was still higher than that in the older cohort. Our rates appeared higher than those previously reported,^55^^,^^56^^,^^57^ which may reflect real-time symptom recording or the misinterpretation of symptoms related to underlying disease. However, by seven days after the second dose, few participants reported residual symptoms. There was a correlation between higher antibodies to RBD after the second vaccine and increased vaccine reactogenicity in both the older and younger cohort. The adverse reactions to the first booster vaccine were similar to those of the initial series in terms of symptoms and severity.

### Strengths

Our unique and nimble study included electronic consent, questionnaires, symptom diaries, and serial self-collected specimens. Our decentralized protocol and recruitment strategy enabled province-wide enrollment, including smaller communities without access to hospital-based research centers. Additional strengths include the cohort size and retention and our ability to quickly adapt to changing vaccine dose and interval recommendations. Other population-based serology studies evaluating vaccine responses through DBS^58^^,^^59^^,^^60^^,^^61^ have small sample sizes, infrequent testing, and younger individuals. Returning individual and group results through the platform kept our population engaged which will contribute to robust long-term evaluation of the serology and the impact of the booster doses. Despite the challenges of rapid implementation of a digital platform, less familiarity of our target population with electronic platforms, tight and changing vaccine distribution timelines, and the pressures of rapid kit distribution and postal services, we have successfully engaged and retained a large cohort of older dedicated participants.

In conclusion, we report on a successful large decentralized research program to study the IgG antibody response to COVID-19 vaccines in a large cohort of ambulatory elderly relative to younger adults. Our work provides data on the age-dependent limitations of antibody responses elicited after the first and second dose of COVID vaccines for a vulnerable group that was underrepresented in the vaccine clinical trials. However, we were able to demonstrate that antibody levels remain high in both age cohorts to 48 weeks after the initial series as a consequence of booster dosing. The rate and severity of breakthrough infections were low. Large prospective population data will provide insight into future vaccine strategies for older adults as the correlates of protective immunity are increasingly understood.

### Limitations of the study

The cohort is less diverse than planned, reflecting the need for English fluency and grasp of web-based technology**.** At 24 and 48 weeks after initial series, the IgG antibody levels to RBD were above the linear range of our assay for 2% and 14% of samples, respectively and 9% of the younger and 15% of the older cohort still had values above the linear range at 48 weeks. Because of the small ceiling effect, we elected to use medians and IQRs when presenting the serology results. Given the small percentage above the linear range for any given time point, knowing their true value would not have changed the medians nor IQRs. When modeling the RBD values, we chose to use the median regression for the same reason. Whether or not seropositivity or a certain level of antibody provides protection against infection or severe disease or hospitalization needs further study. Consequently, the differences in antibody levels we demonstrated between the cohorts and with vaccine brand and underlying disease may not be clinically relevant. Further research is needed on the requirement or the frequency of further vaccine boosters. Use of DBS enabled us to collect specimens at home but limits our ability to study the multifaceted immune response that importantly includes memory B cells and T cell responses, which may further help in the understanding of the correlates of protection. Our breakthrough infection rate was low especially in the older cohort, but it is unclear whether this relates to antibody levels, better adherence to masking and other public health measures, or a combination of factors.

## STAR★Methods

### Key resources table

**Table:** 

REAGENT or RESOURCE	SOURCE	IDENTIFIER
**Antibodies**		

Anti-human IgG#5 secondary antibody fused to horseradish peroxidase (HRP)	Colwill K, et al. A scalable serology solution for profiling humoral immune responses to SARS-CoV-2 infection and vaccination. Clin Transl Immunology. 2022 Mar 23; 11(3):e1380. https://doi.org/10.1002/cti2.1380.^36^ eCollection 2022. PMID: 35356067.	IgG#5-HRP
Anti-RBD IgG antibody VHH72	Colwill K, et al. A scalable serology solution for profiling humoral immune responses to SARS-CoV-2 infection and vaccination. Clin Transl Immunology. 2022 Mar 23; 11(3):e1380. https://doi.org/10.1002/cti2.1380.^36^ eCollection 2022. PMID: 35356067.	VHH72-hFc1X7
Anti-nucleocapsid IgG antibody HC2003	Genscript	Cat#A02039

**Biological samples**		

Whatman DBS cards	Whatman products (Cytiva)	Protein saver card 903
Desiccant	Whatman products (Cytiva)	Dessicant Pack Tyvek 1G DNA sample
Lancets		PRES-ACTV 21GAX1.8mm blue 21GAX2.2mm org
Kit assembly	Marketing Kitchen Janice Dumphie	www.marketingkitchjen.ca

**Chemicals, peptides, and recombinant proteins**		

Full-length spike trimer	Colwill K, et al. A scalable serology solution for profiling humoral immune responses to SARS-CoV-2 infection and vaccination. Clin Transl Immunology. 2022 Mar 23; 11(3):e1380. https://doi.org/10.1002/cti2.1380.^36^ eCollection 2022. PMID: 35356067.	SMT1-1
Receptor binding domain (RBD)	Colwill K, et al. A scalable serology solution for profiling humoral immune responses to SARS-CoV-2 infection and vaccination. Clin Transl Immunology. 2022 Mar 23; 11(3):e1380. https://10.1002/cti2.1380.^36^ eCollection 2022. PMID: 35356067.	RBD (331–521)
Nucleocapsid	Colwill K, et al. A scalable serology solution for profiling humoral immune responses to SARS-CoV-2 infection and vaccination. Clin Transl Immunology. 2022 Mar 23; 11(3):e1380. https://doi.org/10.1002/cti2.1380.^36^ eCollection 2022. PMID: 35356067.	NCAP-1
Blocker BLOTTO	ThermoFisher Scientific	Cat#37530
SuperSignal ELISA Pico Chemiluminescent Substrate	ThermoFisher Scientific	Cat#37069

**Deposited data**		

CITF database	McGill University	Communitytaskforce.ca portal.mchi.mcgill.ca

**Software and algorithms**		

Statistical analysis	R version 4.1.1	R Foundation

**Other**		

Consent video	stopCov website	www.stopcov
DBS video	stopCov website	www.stopcov
DBS manual	stopCov website	www.stopcov

### Resource availability

#### Lead contact

Further information and requests should be directed to and will be fulfilled by the lead contact, Sharon Walmsley (sharon.walmsley@uhn.ca).

#### Materials availability

This study did not generate new unique reagents.

### Experimental model and subject details

#### Experimental model

A decentralized longitudinal cohort study planned to follow participants with two COVID-19 vaccine doses for 48 weeks. A study extension invited participants to be followed for 96 weeks after the initial vaccine series. We report results to 48 weeks post the second vaccine dose. The full protocol is available on the study website www.stopcov.ca. Trial registration: Clinicaltrials.gov. NCT05208983.

### Subject details

#### Recruitment

A data sharing agreement with the Ontario Ministry of Health enabled us to send study information emails to persons receiving the COVID-19 vaccine at an Ontario distribution center who consented to contact for research. A similar e-mail was sent to Ontario Canadian Association of Retired Persons members (www.carp.ca). Participants could enroll through the website prior to the first or second vaccine dose. Participants could enroll if they were ≥70 years or 30–50 years of age. We planned for enrollment of more participants in the older cohort. The only other exclusion criteria was an inability to complete the study documents in English. The subject numbers and demographic characteristics are noted in Table 1.

**Electronic consent** of the initial and study extension including the request to share core data elements with the CITF was completed by each participant on the study website. A video and periodic questions were added to enhance comprehension. The study and electronic consent process were approved by the University Health Network (UHN) Ethics Review Committee. Consented participants used the study website with their personal identification (ID) number and password as a portal for communication with study staff, data entry and for receipt of results. A schedule for required activities on paper and in the portal and e-mail reminders were provided.

### Method details

#### Participant questionnaires

Self-administered electronic questionnaires collected baseline demographic and health data that were stored in an administrative database. Electronic diaries collected data on vaccine date and brand and local and systemic adverse events for 7-day after the first three vaccine doses. Monthly check-in questionnaires captured persistent vaccine related adverse events, booster doses date and brand and new COVID-19 diagnoses.

#### Dried blood spot (DBS) specimens

Protocols for self-collection and shipping of DBS specimens were adapted from those previously shown feasible.^62^ A commercial company (Market kitchen) prepared and distributed the kits which consisted of lancets, dried blood spot cards, desiccant, alcohol swabs, gauze, bandages and envelops for return with affixed postage. Instructions were provided in hard copy and in video on the website. Samples were requested +/−7 days of initial vaccine, three weeks (+/−1 week) after the first vaccine dose, two weeks (+/−1 week) after the second vaccine dose and then every 12 weeks (+/−3 weeks). If the dose interval exceeded 28 days, an additional sample was collected prior to the second vaccine dose. Additional DBS were requested 3–4 weeks after vaccine boosters. Whole blood was collected on Whatman 903 cards using a lancet for finger-prick and dried for a minimum of 2 h before mailing in the regular post. The participant was identified by a bar code on the DBS card. Participants were requested to record the date of collection on the DBS card, and in the portal and the date of mailing in the portal.

### Data sharing agreement

We have a data sharing agreement with the Public Health Agency of Canada, one of our funders, as follows: We have transferred relevant anonymized study data as available to the Canadian COVID-19 Immunity Task Force (CITF) as part of a standard data sharing requirement. This is submitted together with a data dictionary defining each field in the set. External researchers will be able to submit a request to the CITF to receive access to all CITF data through their data access committee. The CITF will employ a rigorous checklist to ensure that these external requests follow all necessary ethical and privacy protocols.

The data provided to the CITF will be stored on the CITF Database. The data on the CITF Database will be held under the custodianship of McGill University or one of its collaborators and be shared via the cloud, both nationally and internationally. Data in the CITF Database can be used by researchers across Canada and in other countries following Data Access Committee (DAC) approval. These transfers will also be made in compliance with Canadian law and research ethics.

A DAC will be responsible for reviewing applications for access to the data and for approving applications that respect the privacy and access policies of the CITF. The DAC will require that researchers confirm that their intended research activities and have received necessary ethics approvals. The data may also be shared with other COVID-19 research databases that follow similar protections and procedures as the CITF Database.

### Method details

#### Laboratory studies

##### Serological assays

Completed DBS cards were mailed to the research unit, checked for quality, registered in a red cap database, and transferred to the Lunenfeld-Tanenbaum Research Institute (LTRI) at Sinai Health for processing (Sinai REB study 21-0112-E). Antibodies were eluted from DBS punches and tested by Enzyme Linked Immunosorbent Assay (ELISA) for antibodies (IgG) against the spike trimer, its receptor binding domain (RBD) and nucleocapsid proteins (NPs) which is previously described in more detail. ^38,51^The ELISA developed in-house on serum and plasma was optimized for sensitivity and specificity parameters. Adaptation to a DBS regimen was performed in collaboration with the National Microbiology Laboratory (NML) who distributed paired plasma and contrived DBS for assay optimization. Correlations in the spike, RBD and nucleocapsid assays between plasma/DBS was >0.95. The DBS assay was validated by Receiver Operating Characteristic (ROC) curve analysis of duplicate samples of positive PCR confirmed cases and negative pre-COVID samples donated by the NML.^38^ As samples with high antibody levels saturate the assays preventing accurate measurement, all samples were tested at two dilutions (primary dilution of 2.5ul of DBS eluate/well (1:4) and a secondary dilution of 0.156 μl/well (1:64)) to ensure a large fraction of the measures would be tested within the linear range of quantification.^51^ We selected to profile total IgG antibodies to the indicated antigens since our results and others^41^ show a strong correlation especially between anti-RBD IgG levels and neutralization titers, enabling us to infer neutralization changes across groups. The vaccines currently secured by the Canadian government are spike-based so reactivity to nucleocapsid should only be from natural infection. Monitoring anti-nucleocapsid antibodies helps to identify possible new or re-infections.

#### Interpretation and reporting of results

Raw values are normalized to a synthetic standard included as a calibration curve on each assay plate to create relative ratios.^51^ For the standard curve, we used recombinant antibodies against RBD (for spike and RBD) and against nucleocapsid. Seropositivity thresholds for each antigen were set at 99% specificity as defined by the ROC curve analysis: relative ratios of 0.482 for anti-spike IgG, 0.324 for anti-RBD IgG and 0.642 for NP using the primary dilution (2.5 μl/well). Specimen overall positivity to vaccine required the IgGs to both spike and RBD to be above the threshold. The seroconversion threshold is the cutoff value mentioned in the text for assigning positivity which is set at 3 SD from the mean of negative controls.

To convert relative ratios to BAU/mL units from DBS, we compared matching DBS and plasma samples (n = 83 for RBD, 97 for NP, 59 for spike). The conversion is a two-step process. The first step applies the formula for conversion of relative ratios to BAU/mL for plasma^51^ with an additional factor of 40 applied to account for the differences in the primary dilution of each sample type (1:160 for plasma, 1:40 for DBS).

Formula 1: log 2 (sample BAU/mL at dilution fold d) = (log_2_(sample RR) - a)/b + log_2_(d∗40)

Where a and b represent the y-intercept and slope of the linear interval of the WHO international standard curve. For NP, a = 0.243 and b = 0.713. For RBD, a = −0.612 and b = 0.766. For Spike, Spike a = 0.604 and b = 0.784.

To keep the same seropositivity threshold in BAU/mL units for DBS and plasma, we applied a log linear conversion factor anchored at log_2_(BAU/ml) = 10 and the seropositivity threshold in BAU/ml as a second step.

Formula 2: log2 (BAU/mL recalibrated @ sample dilution d) = c + d ∗ log2(BAU/mL from formula 1)

For NP, c = −2.497 and d = 1.250. For RBD, c = −2.612 and d = 1.261. For Spike c = −3.575 and d = 1.358.

Antibody levels were also compared with the median levels of convalescent serum obtained 21–115 days after symptom onset in patients with COVID-19 (n = 211 for NP and RBD analyzed at 1:160 and 1:640, n = 80 (subset of the 211) for spike analyzed at 1:160 and 1:2560). [36,41]

### Quantification and statistical analysis

#### Sample size considerations

A sample size of 768 older and 192 younger participants was planned to enable detection of differences in relative ratios of anti-spike and anti-RBD IgG of 0.092 and 0.124 at 48 weeks post second vaccination, with 80% power and a significance level of 0.05, assuming SDs of 0.406 and 0.55 respectively based on our data from a study from our group of the elderly in long term care. We anticipated 25% dropout post second vaccination (through attrition or poor quality DBS) and adjusted our target sample size to 1286.

#### Statistical analysis

Baseline characteristics and vaccine type were summarized by age group using median (Interquartile Range, IQR) for continuous variables and count (percent, %) for categorical variables. Adverse events and antibody levels were compared by age group, vaccine type, and reactogenicity using chi-square, Fisher’s Exact, and Kruskal-Wallis tests. Demographic and clinical data were compared by serostatus for anti-RBD IgG at 2 weeks post second dose using Fisher’s Exact test. Univariable and multivariable median regression were used to model antibody levels to RBD in BAU/mL at 2, 24, and 48 weeks after the second vaccine dose (separately) and test for an association with age. Covariates included in the multivariable models were chosen *a priori* based on clinical expertise and included gender, comorbidities (cardiovascular disease (CVD), cancer, diabetes, immunosuppression), body mass index (BMI), previous COVID infection (defined as a positive nucleoprotein (NP) antibody, or self-reported positive COVID PCR or rapid antigen test; RAT), booster doses, and receipt of mRNA-1273. The threshold for significance was defined as 0.05. R version 4.1.1 was used for all analyses.

## Data Availability

The data supporting the findings of this study are available within the paper and the raw data available from the lead contact upon request and with the appropriate and approved data sharing agreement. De-identified data have been shared and deposited with the CITF. portal.mchi.mcgill.ca(See data sharing agreement below for instructions for access). We have converted our antibody results to WHO binding antibody units (BAU)/mL units to allow comparison with other studies. Any additional information required to reanalyze the data reported in this paper is available from the lead contact upon request. The statistical code used to clean, format and analyze the data is available from the lead contact on request. It has not been deposited in a publicly available site. The study protocol, statistical analysis plan, informed consent form and full protocol are available on the study website www.stopcov.ca. Any additional information required to reanalyze the data reported in this paper is available from the lead contact on request. The web-based platform and software was developed by The Data Aggregation, Translation, and Architecture (DATA) team at the University Health Network. Further information on its structure can be made available from the lead author and with the appropriate data sharing agreement.
